# Antibody persistence of standard versus double three-dose hepatitis B vaccine in liver transplant children: a randomized controlled trial

**DOI:** 10.1038/s41598-024-51149-w

**Published:** 2024-01-04

**Authors:** Palittiya Sintusek, Supranee Buranapraditkun, Siriporn Khunsri, Warunee Polsawat, Preeyaporn Vichaiwattana, Yong Poovorawan

**Affiliations:** 1 Center of Excellence in Thai Pediatric Gastroenterology, Hepatology and Immunology (TPGHAI), Department of Pediatrics, Faculty of Medicine, King Chulalongkorn Memorial Hospital and the Thai Red Cross Society, Chulalongkorn University, Bangkok, 10330 Thailand; 2 Division of Allergy and Clinical Immunology, Department of Medicine, King Chulalongkorn Memorial Hospital, Faculty of Medicine, Chulalongkorn University, Bangkok, 10330 Thailand; 3 Excellence Center for Organ Transplantation, King Chulalongkorn Memorial Hospital and the Thai Red Cross Society, Bangkok, 10330 Thailand; 4 Excellence Center of Clinical Virology, Department of Pediatrics, Faculty of Medicine, King Chulalongkorn Memorial Hospital, Chulalongkorn University, Bangkok, 10330 Thailand

**Keywords:** Immunology, Gastroenterology

## Abstract

Rapid hepatitis B (HB) surface antibody (anti-HBs) loss is prevalent after liver transplantation (LT). Herein, we evaluated anti-HBs persistence after HB vaccination using two regimens in LT children. We recruited 66 previously immunized LT children with anti-HBs level of < 100 mIU/mL. Participants were randomly reimmunized with standard-three-dose (SD) and double-three-dose (DD) intramuscular HB vaccination at 0, 1, and 6 months. Anti-HBs were assessed at every outpatient visit. Antibody loss defined as anti-HBs levels < 100 mIU/mL after three-dose vaccination. After three-dose vaccination, 81.8% and 78.7% of participants in the SD and DD groups, had anti-HBs levels > 100 mIU/mL, with a geometric mean titer (GMT) of 601.68 and 668.01 mIU/mL (*P* = 0.983). After a mean follow-up of 2.31 years, the anti-HBs GMT was 209.81 and 212.61 mIU/mL in the SD and DD groups (*P* = 0.969). The number of immunosuppressants used and an anti-HBs level < 1 mIU/mL at baseline were independently associated with anti-HB loss. The DD regimen strongly increased the risk of anti-HBs loss (adjusted hazard ratio, 2.97 [1.21–7.31]; *P* = 0.018). The SD HB reimmunization regimen effectively maintained protective anti-HBs levels in children undergoing LT, making it the preferred regimen for such children with anti-HB loss.

Trial registration: TCTR20180723002.

## Introduction

Hepatitis B (HB) virus is the leading cause of chronic hepatitis^1–4^, and vaccination is considered a crucial, high-efficacy HB infection prevention strategy^5^. While immune protection does not depend on the level of antibodies in immunocompetent hosts^6^, antibody loss in immunocompromised patients may reflect a loss of this protection^7–9^. In the liver transplant (LT) setting, de novo hepatitis B infection (DNH) was observed in previously-immunized children who underwent LT^8^. Hence, a high HB surface antibody (anti-HBs) level is a correlate of immunity and offers the simplest way to demonstrate durable protection in this vulnerable group. As a result, many pediatric LT centers follow a booster dose policy to help patients maintain high anti-HB levels and prevent DNH^10–14^. Booster doses are more cost-effective than the administration of HB immune globulin or antiviral agents^14^. However, the persistence of anti-HBs is closely related to the peak anti-HB response, and the anti-HBs concentration declines quickly after a booster dose^15^. Even with the frequent use of booster HB vaccines to maintain a high anti-HBs level, children who have undergone LT may be diagnosed with DNH^8,10,11,14,16^.

In addition to frequent booster doses, strategies to increase the efficacy of HB vaccines to maintain high anti-HBs levels include multiple doses at appropriate intervals, intradermal vaccination, high dose vaccinations, and using an appropriate type of vaccine or adjuvant^17^. Meta-analyses and guidelines recommend the use of a double dose of three-dose HB vaccine in patients on hemodialysis^18^ and in those positive for human immunodeficiency virus (HIV)^19,20^. Few prospective studies have compared the efficacy of multiple-dose and double-dose HB regimens between adults and children with liver diseases or LT^21–24^ who show an unsatisfactory antibody response. Recently, we conducted a randomized controlled trial (RCT) to compare the standard three-dose (SD) and double three-dose (DD) HB vaccination regimens (at 0, 1, and 6 months) in previously immunized children who had undergone LT and had anti-HB loss. We found that the anti-HBs level was significantly higher, at > 100 mIU/mL, and was more persistently maintained after three-dose HB immunization than after a booster dose in a short-term follow-up (199 days after completion of the 3-dose HB revaccination)^25^. Thus, in the present study, we aimed to compare the long-term HB immunity obtained after completion of the SD and DD regimens for HB vaccination in children who had undergone LT. Factors associated with the loss of HB immunity over time were also assessed.

## Methods

### Study design and participants

This prospective study was based on our previous study^25^, which was approved by the Institutional Review Board of Chulalongkorn University (IRB No.142/60) and registered in the Thai Clinical Trials Registry (TCTR20180723002) on 23/07/2018. All research was conducted in accordance with both the Declarations of Helsinki and Istanbul. Written informed consent and/or assent for the participation and publication of their details was obtained from the parents of all the children’s and/or from participants.

**Figure 1:**
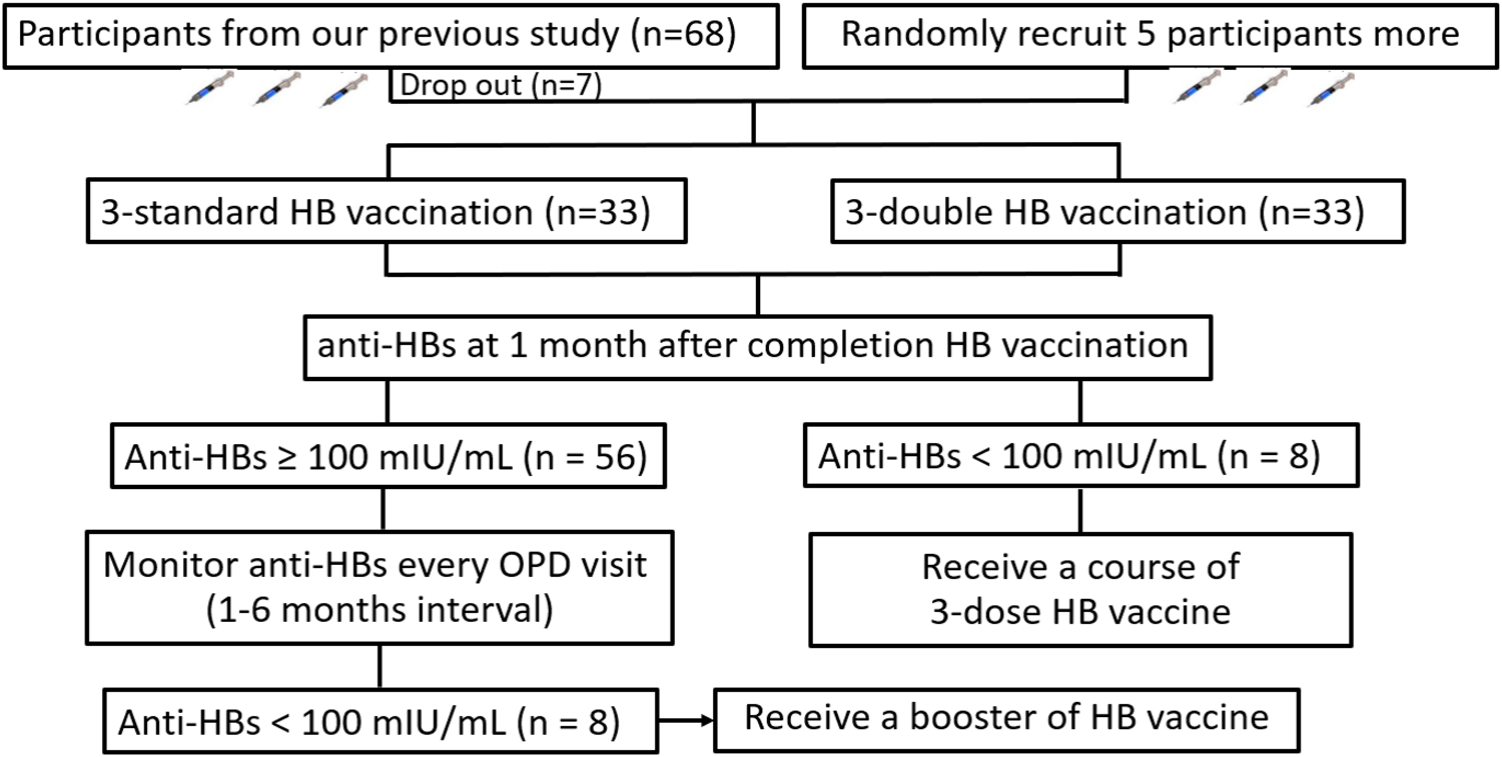
Analysis population and patient flow.

In brief, 61 children who underwent LT at King Chulalongkorn Memorial Hospital, Bangkok, Thailand, from September 2017 to June 2021 were randomly allocated in blocks of 4 at a 1:1 ratio to receive either a standard 3-dose HepB vaccine (SD) or double 3-dose HepB vaccine (DD). Additionally, we randomly enrolled five more participants in the present study. Thus, in this study, 66 participants randomly received the three-dose HB vaccine (rDNA, GlaxoSmithKline, Belgium) at standard (10 µg) and double (20 µg) doses at 0, 1, and 6 months. After completion of a three-dose HB vaccine, the anti-HBs level was evaluated during an outpatient visit every 1–6 months depending on each patient’s follow-up period. The follow-up period was determined by the doctor-in-charge based on previous anti-HBs values. Patient demographic and clinical characteristics were recorded. Anti-HBs were measured by an automated enzyme-linked immunosorbent assay performed using the ARCHITECT system (Abbott, Wiesbaden, Germany) according to the manufacturer’s instructions, with a cut-off point of > 1 mIU/mL. Children with an anti-HBs level < 100 mIU/mL during the follow-up period received one booster dose of the HB vaccine (0.5 mL). Children with an anti-HBs level of < 10 mIU/mL after completion of the three doses received a course of three-dose HB revaccination (Fig. 1).

### Definitions

The protective level of anti-HBs in LT children in the present study was ≥ 100 mIU/mL^25^. Thus, anti-HBs loss was defined as an anti-HBs level of < 100 mIU/mL during the follow-up period. The follow-up duration was defined as the time from completion of the three-dose HB revaccination to the censoring date.

### Statistical analysis

Continuous data are presented as mean (standard deviation) or median (interquartile range) values based on the distribution of variables. Categorical data are presented as numbers and percentages. The unpaired *t*-test and Mann–Whitney *U* test were used to compare the data, as appropriate. Discrete data were compared using the chi-square test or Fisher’s exact test, as appropriate. The geometric mean titer (GMT) was calculated based on an anti-HBs titer of > 1 mIU/mL and represented logarithmically. The failure rate or anti-HB loss rate was assessed using Kaplan–Meier analysis. The anti-HB loss rate was compared between the SD and DD groups using a log-rank test. Parameters that might be associated with anti-HB loss were analyzed using univariate and multivariate Cox proportional hazards regression analyses. Data analysis was performed using Stata version 17 (Stata Corp, LLC, College Station, TX, USA). Statistical significance was set at *P* < 0.05. This study was reviewed by a biomedical statistician from the Department of Statistical Science, Kasertsart University, Thailand.

## Results

### Patient characteristics

Of the 73 children included in the study, seven dropped out during the three-dose HB revaccination period. Each group (SD and DD) included 33 participants, with no statistically significant between-group differences in baseline characteristics (Table 1). The median age of participants who received a three-dose HB revaccination course was 4.08 (1.73–9.69) years, and 53% were female. The mean anti-HBs level before vaccination was 13.63 (23.47) mIU/mL. The most common indication for LT was biliary atresia (77.3%). Tacrolimus was the most commonly administered immunosuppressive agent (69.7%). Eight, 38, and 10 children who had undergone LT received two, three, and four HB vaccine injections, respectively, before LT. Only two children received anti-HBc-positive liver grafts from donors.

**Table 1:** Patient demographics and characteristics at baseline by vaccination arm. *P-values obtained using the Mann–Whitney U and Chi-square tests. *Anti-HBc* hepatitis B core antibody, *Anti-HBs* hepatitis B surface antibody, *GGT* gamma glutamyl transpeptidase, *GMT* geometric mean titer, *HB* hepatitis B, *Hb* Hemoglobin, *AST* serum aspartate aminotransferase, *ALT* serum alanine aminotransferase, *LT* liver transplant, *WBC* white blood cell count.

Variables	Standard dose (n = 33)	Double dose (n = 33)	*P*-value*
Age at LT (years)	1.2 (0.85, 2.87)	1.5 (0.97, 3.67)	0.352
Male (n, %)	14 (42.4%)	17 (51.5%)	0.459
Age at vaccination (years)	3.92 (1.73, 7.83)	4.97 (2.17, 10.9)	0.485
Time from LT to vaccination (years)	1.31 (0.73, 3.88)	1.35 (0.64, 4.24)	0.715
Underlying disease—biliary atresia	26 (78.8%)	25 (75.8%)	0.769
Anti-HBc status—positive	1 (3%)	1 (3%)	1
Number of HB vaccine before LT (years)			
1	2 (6.1%)	0 (0%)	0.086
2	6 (18.2%)	6 (18.2%)	
3	16 (48.5%)	24 (72.7%)	
4	9 (27.3%)	3 (9.1%)	
Number of immunosuppressants used			
0	1 (3%)	0 (0%)	0.662
1	14 (42.4%)	15 (45.5%)	
2	14 (42.4%)	12 (36.4%)	
3	4 (12.1%)	6 (18.2%)	
Type of immunosuppressant used			
Tacrolimus	24 (72.7%)	30 (90.9%)	0.139
Cyclosporin	8 (24.2%)	3 (9.1%)	
None	1 (3%)	0 (0%)	
Level of immunosuppressants			
Tacrolimus (ng/mL)	3.3 (2.45, 5.25)	3.9 (3.3, 5.6)	0.292
Cyclosporin (ng/mL)	223 (166, 442.5)	386 (118, 835)	0.734
Laboratory investigations			
AST (IU/L)	41 (35, 48)	46 (34, 55)	0.323
ALT (IU/L)	31 (21, 38)	32 (23, 56)	0.724
GGT (IU/L)	24 (17, 70)	35 (23, 146)	0.088
Albumin (g/dL)	4.2 (4, 4.4)	4.1 (3.9, 4.2)	0.188
Hb (g/dL)	11.9 (10.7, 12.7)	11.2 (10, 12)	0.069
WBC (× 10^9^ L)	7.85 (6.45, 11.59)	8.31 (6.35, 9.76)	0.509
Neutrophil count (× 10^6^/L)	3420 (2830, 4220)	3290 (2560, 4990)	0.99
Lymphocyte count (× 10^6^/L)	1100 (288, 6120)	2630 (501, 4740)	0.883
Platelet count (× 10^9^)	244 (206, 303)	228 (184, 294)	0.568
Anti-HBs level (mIU/mL) at baseline	1.7 (0.1, 7.4)	5.8 (1.2, 14)	0.069
Anti-HBs level at baseline (n, %)			
< 10 mIU/mL	14 (42.4%)	8 (24.2%)	0.179
10–100 mIU/mL	12 (36.4%)	12 (36.4%)	
> 100 mIU/mL	7 (21.2%)	13 (39.4%)	
Antibody GMT			
Anti-HBs level at the end of follow-up period (mIU/mL)	301 (68, 653.69)	94.3 (63.7, 1297.68)	0.793
Follow-up time (years)	2.78 (1.28, 3.93)	1.86 (0.36, 3.2)	0.099
Patients with an anti-HBs level < 100 mIU/mL at the end of the follow-up period (n, %)	12 (36.4%)	18 (54.5%)	0.138

### Persistence of anti-HBs level after completion of the three-dose HB vaccination

The rates of anti-HBs ≥ 100 mIU/mL 1 month after complete revaccination were 81.8% and 78.7% in the SD and DD groups, respectively. The anti-HBs GMT was 601.68 (95% confidence interval [CI] 214.64–1686.69) and 668.01 (95% CI 268.99–1658.94) mIU/mL in the SD and DD groups, respectively (*P* = 0.983). At 2.31 (0.99–3.64) years from completion of the three-dose HB revaccination schedule, 75.0% and 60.7% of participants had an anti-HBs level of > 100 mIU/mL in the SD and DD groups, respectively (*P* = 0.391). The follow-up duration (2.67 [2.11–3.22] vs 2.01 [1.46–2.54], *P* = 0.086) and the rate of antibody loss over time (*P* = 0.100) did not differ between the SD and DD groups. At the end of the follow-up period, the anti-HBS GMT was 209.81 (95% CI 100.82–436.63) and 212.61 (95% CI 102.02–443.06) mIU/mL in the SD and DD groups, respectively (P = 0.969).

**Figure 2:**
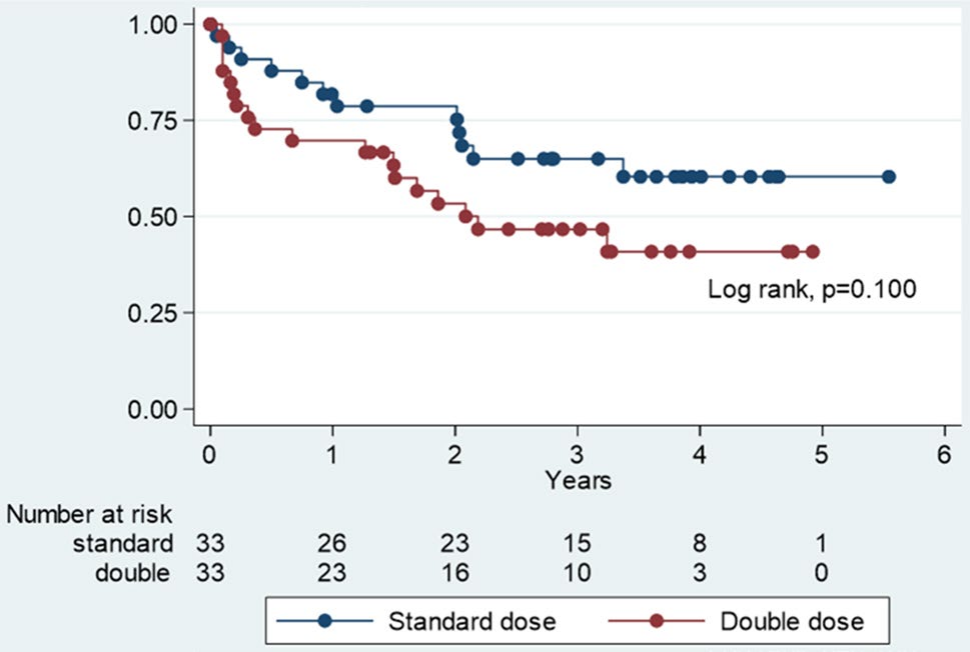
Comparison of estimated anti-HBs persistence rates between the standard and double-dose groups.

Kaplan–Meier analysis revealed that anti-HBs loss took longer in the SD group. Anti-HB persistence rates are listed in Table 2. Estimated anti-HBs persistence rates were 81.82% (95% CI 63.94–91.39%) vs 69.7% (95% CI 51.01–82.4) at 1 year and 60.39% (95% CI 40.3–75.53%) vs 40.83% (95% CI 22.48–58.42%) at 4 years in the SD and DD groups, respectively (Fig. 2).

**Table 2:** Anti-HBs persistence rate (anti-HBs level > 100 mIU/mL) in the standard and double dose groups. *Anti-HBs* hepatitis B surface antibody, *CI* confidence interval.

Years	Standard dose (n = 33)		Double dose (n = 33)	
	Anti-HBs level > 100 mIU/mL (%)	95% CI	Anti-HBs level > 100 mIU/mL (%)	95% CI
1	81.82	63.94–91.39	69.7	51.01–82.4
2	78.67	60.39–89.22	53.33	34.79–68.78
3	64.99	45.59–78.94	46.67	28.75–62.75
4	60.35	40.3–75.53	40.83	22.48–58.42
5	60.35	40.3–75.53	–	–

### Cox regression analysis of factors associated with anti-HBs loss

**Figure 3:**
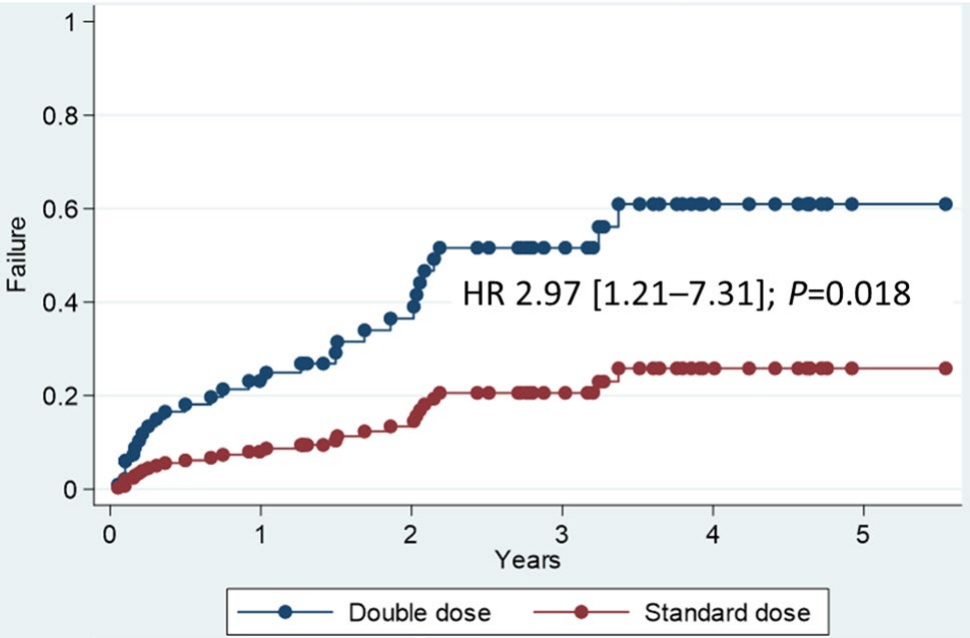
Kaplan–Meier analysis under cox proportional hazard regression model demonstrates the significantly longer rate of anti-HBs loss in SD group compared with DD group. Adjusted for group, sex, age at vaccination, time from LT to vaccination, anti-HBs at baseline, and number of IMs used.

Univariate Cox regression analysis revealed an association between anti-HBs loss over time, age at vaccination, and time from LT to vaccination (Table 3). The analysis also supported an increased risk of anti-HB loss over time in children who had undergone LT and received multiple immunosuppressants (IMs). Multivariate Cox regression analysis revealed that the number of IMs used, anti-HBs level < 1 mIU/mL before vaccination, and double-dose vaccination (Table 3) were associated with anti-HB loss with time. The number of IMs was strongly associated with an increased risk of anti-HBs loss over time. Administration of two and three IMs had adjusted hazard ratios (HRs) of 4.86 (95% CI 1.64–14.45; *P* = 0.004) and 17.65 (9%% CI 3.96–78.68; *P* < 0.001), respectively. An anti-HBs level of < 0.1 mIU/mL before vaccination was strongly associated with an increased risk of anti-HB loss over time (HR, 3.85 [1.08–13.65]). Participants in the DD group also had an increased risk of anti-HBs loss over time (HR, 2.97 [1.21–7.31]; *P* = 0.018) (Fig. 3).

**Table 3:** Median time for anti-HBs loss categorized based on potential parameters and patient’s characteristics. *P-values obtained using the log-rank test. *Anti-HBs* hepatitis B surface antibody, *CI* confidence interval, *HR* hazard ratio, *IM* immunosuppressant, *LT* liver transplant.

Parameters	Univariate analysis			Multivariate analysis		
	Crude HR	95% CI	*P*-value	Adjusted HR	95% CI	*P*-value
Group						
Standard dose	Ref	1	1	Ref	1	1
Double dose	1.83	0.88–3.81	0.106	2.97	1.21–7.31	0.018*
Sex						
Male	0.85	0.41–1.74	0.648	1.18	0.47–2.97	0.764
Female	Ref	1	1	Ref	1	1
HB vaccine before LT (times)						
1	1.68	0.20–14.48	0.636	0.67	0.05–9.38	0.764
2	1.95	0.62–6.19	0.254	0.74	0.15–3.52	0.700
3	1.12	0.41–3.05	0.817	0.30	0.78–1.18	0.085
4	Ref	1	1	Ref	1	1
Age at vaccination (years)						
< 5	Ref	1	1	Ref	1	1
5–10	0.22	0.05–0.96	0.044*	0.56	0.09–3.58	0.542
> 10	1.35	0.62–2.93	0.445	1.49	0.49–4.54	0.477
Time from LT to vaccination (years)						
≤ 3	3.82	1.44–10.11	0.002*	3.85	0.99–15.03	0.052
> 3	Ref	1	1	Ref	1	1
Anti-HBs level baseline (mIU/mL)						
< 1	1.53	0.58–4.02	0.390	3.85	1.08–13.69	0.037*
1–10	1.53	0.61–3.83	0.367	0.93	0.29–2.93	0.899
10–100	Ref	1	1	Ref	1	1
Number of IMs used						
0–1	Ref	1	1	Ref	1	1
2	3.5	1.36–9.04	0.01*	4.68	1.58–13.87	0.005*
3	8.87	3.1–25.33	< 0.001*	12.55	3.17–49.71	< 0.001*

## Discussion

This study demonstrated the efficacy of SD and DD HB vaccination during long-term follow-up in children who had undergone LT. In the SD and DD groups, 60.4% and 41% of participants, respectively, had persistent protective levels of anti-HBs (> 100 mIU/mL) at the 4-year follow-up, respectively. Multivariate Cox regression analysis showed that among children who had undergone LT, the use of multiple IMs and an anti-HBs level of < 1 mIU/mL before vaccination were strongly associated with an increased risk of anti-HB loss over time. In addition, the DD regimen was an independent factor that increased the risk of anti-HBs loss over time.

To the best of our knowledge, the present study is the first RCT to compare the effectiveness of the SD and DD vaccines (at 0, 1, and 6 months) in previously immunized children who had undergone LT and experienced anti-HB loss. Our previous study, which had a short-term follow-up duration, demonstrated that complete SD or DD HB vaccination helped maintain a higher anti-HB level than after a booster HB vaccine dose^25^. The antibody response after completion of the DD HB vaccine regimen tended to be lower than that after completion of the SD regimen in our previous study. Similarly, the multivariate Cox analysis in the present continuous study revealed that among children who had undergone LT, the DD HB vaccine regimen carried a higher risk of anti-HBs loss over time.

Vaccination is the most effective interventional therapy for controlling infectious diseases. However, vaccine effectiveness is adversely affected by repeated vaccinations^26–28^, immune imprinting^29,30^, pre-existing anti-vector immunity^31^, short intervals between repeated vaccines^32,33^, and the co-administration of multiple vaccines^34^. Immune interference is the common phenomenon in influenza vaccine and coronavirus disease 2019 vaccine because of their variable antigenic sites in viral proteins. Immune imprinting of previous vaccinations and mismatching between viral strains and vaccine strains are the explanation of the vaccine ineffectiveness with the repeated vaccination^35^. Unlike that for the influenza and the coronavirus disease 2019 vaccines, there is limited evidence supporting immune interference with the HB vaccine. Fonzo et al*.* demonstrated that delaying HB vaccine administration within the first year of life could affect the long-term maintenance of anti-HBs levels. Each month of delay within the first year of life was associated with a 16% reduction in maintaining an anti-HB titer of < 10 IU/mL approximately 20 years after primary vaccination^36^. Impaired T-cell function and the fewer interactions between B and T cells in infants^37,38^ necessitate repeated antigenic administrations, and a longer interval between repeat vaccine administration effectively maintains the appropriate anti-HBs level. However, the mechanism behind the antigenic overload that might interfere with the immune response to the HB vaccine has not been documented so far. Double dose regimen for hepatitis B vaccine was effective in HIV-infected patients as higher immunogenicity was observed, when it was measured 4–6 weeks and > 12 months after completion of the vaccination compared with standard dose regimen^20^. The conflict result of the anti-HBs response to double dose HB vaccine regimen from the present study and the study in HIV-infected patients might be explained by the complexity of cellular immune interactions after revaccination at higher doses in different immunocompromised patients that requires further investigation.

This study also identified the use of multiple IMs and anti-HB levels of < 1 mIU/mL before revaccination as other factors strongly associated with anti-HB loss over time. Although B cells are key mediators of rigorous immunity that prevent HB infection, T cells play a vital role in stimulation B-cells for antibody production^39^. HIV positivity and a low CD4 count are examples of T cell defects leading to poor anti-HB response and long-term persistent immunity after revaccination^40^. In our previous study, using an enzyme-linked immunosorbent spot assay to evaluate the cellular immune response to the HB vaccine, we demonstrated a significant T-helper1 cell response with significantly higher-secreting cells in responders than in hyporesponders^25^, which was in line with the findings of Ni et al*.*^13^. In addition to T and B cell responses after vaccination, immune memory should be sufficiently rigorous to protect against pathogens. Ample evidence shows waning immunity with a rapid anamnestic response in healthy individuals with an anti-HBs level of < 10 mIU/mL, do not require revaccination^6^ whereas anti-HBs loss might reflect immunity loss as evidenced by DNH in LT children. The present study found that the cutoff anti-HBs level of < 1 mIU/mL was significantly associated with the rate of anti-HB loss over time after HB vaccination. The low quantity or quality of immune memory cells in participants with very low anti-HBs levels might explain the increased risk of anti-HBs loss over time. This merits further investigation. In terms of clinical implications, while the number of IMs required depends on the patient’s condition after LT and may not be modified, increasing anti-HB levels before revaccination after LT could be improved by administering a booster dose to children waiting for LT. Generally, the anti-HBs level will decrease with time after LT, but a booster dose of HB vaccination before LT would ensure that the anti-HBs level remains adequate to prevent DNH during 6-month post-LT. After that, monitoring anti-HBs level and revaccination with 3-dose HB vaccine when anti-HBs level declines to less than 100 mIU/mL. We suggest that this strategy be used for increasing and maintaining a high anti-HBs level during long-term follow-up to guarantee protection against HB infection in children who undergo LT.

A limitation of this study is that we included only a few cases of LT with anti-HBc-positive liver grafts (n = 2). Such patients have a high risk of DNH, and this requires a separate subgroup evaluation, as their immune response after vaccination might differ from that of children who undergo LT with anti-HBc-negative liver grafts. Furthermore, the role of the cellular immune response in the persistence of anti-HBs during long-term follow-up was not evaluated in this study. Last, the power of this study is low because of the small number of recruited participants that might lead to statistical insignificance of the main primary outcome or the rate of anti-HBs loss overtime comparing between SD and DD regimen. However, according to multivariate Cox regression analysis that included the clinically significant parameters in the data analysis, we found that DD was an independent factor associated with anti-HBs loss overtime. This result might imply the less effectiveness of DD regimen comparing to SD regimen in LT children that deserves further large clinical trials to confirm it.

In summary, the SD regimen for HB reimmunization demonstrated high effectiveness in maintaining a protective level of anti-HBs in children undergoing LT. Therefore, it is recommended that children who have undergone LT and experience anti-HBs loss should be scheduled for the SD regimen to ensure sustained protection.

## Data Availability

Requests for materials should be addressed to P.S.
